# The Origin and Biomedical Relevance of Cannabigerol

**DOI:** 10.3390/ijms23147929

**Published:** 2022-07-19

**Authors:** Anna Jastrząb, Iwona Jarocka-Karpowicz, Elżbieta Skrzydlewska

**Affiliations:** Department of Analytical Chemistry, Medical University of Bialystok, A. Mickiewicza 2D, 15-222 Bialystok, Poland; anna.jastrzab@umb.edu.pl (A.J.); iwona.jarocka-karpowicz@umb.edu.pl (I.J.-K.)

**Keywords:** *Cannabis sativa* L., cannabigerol-type group, cannabigerol, biosynthesis, biological activity, pharmacokinetics

## Abstract

The constant search for new pharmacologically active compounds, especially those that do not exhibit toxic effects, intensifies the interest in plant-based ingredients and their potential use in pharmacotherapy. One of the plants that has great therapeutic potential is *Cannabis sativa* L., a source of the psychoactive Δ^9^-tetrahydrocannabinol (Δ^9^-THC), namely cannabidiol (CBD), which exhibits antioxidant and anti-inflammatory properties, and cannabigerol (CBG)—a biologically active compound that is present in much smaller quantities. CBG is generated during the non-enzymatic decarboxylation of cannabigerolic acid, a key compound in the process of biosynthesis of phytocannabinoids and consequently the precursor to various phytocannabinoids. By interacting with G-protein-coupled receptors, CBG exhibits a wide range of biological activities, inter alia, anti-inflammatory, antibacterial and antifungal activities, regulation of the redox balance, and neuromodulatory effects. Due to the wide spectrum of biological activities, CBG seems to be a very promising compound to be used in the treatment of diseases that require multidirectional pharmacotherapy. Moreover, it is suggested that due to the relatively rapid metabolism of cannabigerol, determination of the concentration of the phytocannabinoid in blood or oral fluid can be used to determine cannabis use. Therefore, it seems obvious that new therapeutic approaches using CBG can be expected.

## 1. Introduction

Recent years have seen a significant increase in the interest in the use of plant-derived ingredients in pharmacotherapy. Although plants and their components have been used in therapeutic activities for several thousand years, their conscious use as elements of drugs, dietary supplements, cosmetics, and other products exhibiting biomedical properties are the result of research carried out over the last 20 years. One of the plants whose ingredients are more and more often studied for use in biomedical and pharmaceutical activities is *Cannabis sativa* L. At the same time, it should be noted that compounds obtained from *Cannabis sativa* L. are usually considered more beneficial than synthetic ones, because the latter may cause unwanted side effects when used for longer periods of time [1].

Consequently, many drugs that are currently clinically investigated contain components of plant origin, whose biological functions may vary. In particular, it has been shown that plant extracts can act, e.g., as antimicrobial agents by significantly reducing the viability of pathogens. In light of the growing problem of antibiotic resistance, this could be of great importance in clinical medicine [2]. Furthermore, some bioactive compounds found in medicinal plants have been shown to exhibit antifungal activity [3]. This has led to an increased interest in their potential use from representatives of both medicine and pharmacy. Another area of interest is the antioxidant properties of medicinal plant-based ingredients, including their ability to scavenge free radicals generated in disease conditions, thus making it possible to enhance the body’s antioxidant capacity [4]. Since antioxidant properties are often accompanied by anti-inflammatory properties, bioactive compounds contained in medicinal plants often also regulate the efficiency of the transcription factor NFκB and, by inhibiting TNFα generation, exhibit anti-inflammatory properties [5]. Consequently, the study of bioactive compounds of plant origin has led to the discovery of drugs with a potential therapeutic value, especially in the treatment of cancer [6].

One of the plants that are being increasingly often studied, due to the promising multidirectional biomedical activity of their constituents, is *Cannabis sativa* L., a plant that has been cultivated since ancient times, mainly for its fibers and oil, but also for its medicinal properties [7]. The plant is a source of many biologically active compounds [8], making it especially interesting as a source of substances already defined as medicinal as well as compounds under investigation as potentially exhibiting medicinal properties [8,9]. Of particular interest are those components of *Cannabis sativa* L. that show antioxidant properties and thus may potentially modulate oxidative stress that accompanies the development of many diseases [9,10].

Among the various groups of constituents of *Cannabis sativa* L., phytocannabinoids, i.e., dibenzopyrene or monoterpenoid derivatives that exhibit a range of activities modulating metabolic changes in the human body, are currently attracting the greatest interest due to their similarities to endocannabinoids—in direct as well as indirect action through G-protein-coupled membrane receptors. Consequently, both single compounds and natural mixtures are increasingly often being tested for their potential medical use.

Currently, attention is focused mostly on phytocannabinoids, which do not exhibit psychoactive activity but beneficially modify cellular metabolism. In terms of potential use in pharmacotherapy, cannabidiol is the one studied the most frequently and extensively and is primarily evaluated for its antioxidant and anti-inflammatory properties [11]. In contrast, the most recent literature data on biomedical activity focuses on another phytocannabinoid, i.e., cannabigerol (CBG) [12], a precursor of other cannabinoids such as cannabidiol (CBD), cannabichromene (CBC), and Δ^9^-tetrahydrocannabinol (Δ^9^-THC) [13].

## 2. *Cannabis sativa* L. as a Source of Cannabigerol

A characteristic feature of cannabis is the large number of trichomes, which take the form of protuberances and cover the plant’s leaves and stems. The trichomes found in *Cannabis sativa* L. can be divided into two types: glandular (secretory) and non-glandular. Within the secretory trichomes, numerous biologically active compounds are biosynthesized and/or secreted, such as terpenoids (responsible for the fragrance of hemp) and phytocannabinoids, whose role is to protect the plant from pests and herbivores. In addition, over 750 compounds with diverse biological activities have been identified in hemp, including flavonoids (23 chemical individuals identified), terpenoids (140 com-pounds), and cannabinoids (86 compounds) [7,8], with the content of the particular chemical individuals closely related to hemp variety. This variation is particularly evident in the content of Δ^9^-THC, which depends on the intended use of the cultivated plants. Cultivars of *Cannabis sativa* L. used for typically industrial purposes (e.g., connected with the textile industry or obtaining construction biomaterials) contain insignificant amounts of the psychoactive cannabinoid, whose high concentrations can be found in the so-called ‘medical varieties’. Connected with this is the fact that cultivation of varieties with significant concentrations of Δ^9^-THC is illegal in many countries—only cultivation for medical and scientific purposes is allowed [14]. Variations in phytocannabinoid contents resulting from the different intended uses of cannabis cultivars also manifest in variable contents of those cannabinoids that do not exhibit psychoactive effects, including cannabidiol and cannabigerol [15]. It is worth noting that the highest contents of CBG within a single plant can be found in those flowers and leaves of inflorescences that are collected from the highest parts of the plant—the contents are approx. 10 times higher than in the case of fan leaves [14].

**Figure 1:**
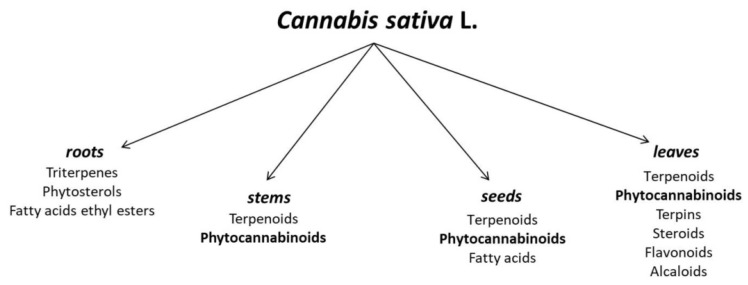
Chemical composition of different parts of *Cannabis sativa* L. [17,18].

Apart from the significant terpenoid and cannabinoid contents, hemp is also a source of such compounds as carbohydrates (mono-, di- and polysaccharides, and amino sugars), flavonoids (e.g., terpinolene, quercitrin, kaempferol), fatty acids (e.g., α-linolenic acid, oleic acid, and linoleic acid), phytosterols, vitamins, and simple alcohols, esters, and organic acids (Figure 1). It is noteworthy that in the case of fatty acids, a total of 33 acids have been identified in hemp seed oil, with unsaturated acids as the clearly dominant group. The oil is a valued source of linoleic (LA), α-linolenic (ALA), oleic (OA), γ-linolenic (GLA), stearidonic (SDA), and cis-vaccenic acids [16].

Phytocannabinoids are a group of 21-carbon terpenophenolic compounds [19]. To date, more than 120 phytocannabinoids have been isolated from cannabis, including two compounds, (−)-trans-Δ^9^-tetrahydrocannabinol (Δ^9^-THC) and (−)-trans-Δ^8^-tetrahydrocannabinol (Δ^8^-THC), which produce the characteristic psychotropic effect by binding to cannabinoid receptors [20]. Another group, containing 16 of the phytocannabinoids, are cannabigerol and its derivatives [21]. Apart from the above phytocannabinoids, hemp contains cannabinol (CBN), cannabidiol (CBD), cannabichromene (CBC), Δ^9^-tetrahydrocannabivarin (THCV), cannabivarin (CBV), and cannabidivarin (CBDV) [22]. Other phytocannabinoids, such as cannabinodiol (CBND), cannabielsion (CBE), cannabicyclol (CBL), and cannabitriol (CBT), have also been the subject of research in recent decades despite their lower contents in *Cannabis sativa* L. [23].

It is believed that, similarly to endocannabinoids, phytocannabinoids also affect the human body through their interaction with G-protein-coupled membrane receptors, including cannabinoid receptors (CB1 and CB2), to which individual members of the group show very different affinities [22]. Furthermore, molecular targets outside the endocannabinoid system have been identified in recent years for some phytocannabinoids. Plant cannabinoids have been shown to interact with other G protein-coupled receptors (GPR55 or GPR18 receptors) and opioid or serotonin receptors, as well as nuclear receptors and ligand-gated ion channels or transient receptor potential (TRP) channels [24].

## 3. Structure of Compounds from the Cannabigerol-Type Group

**Figure 2:**
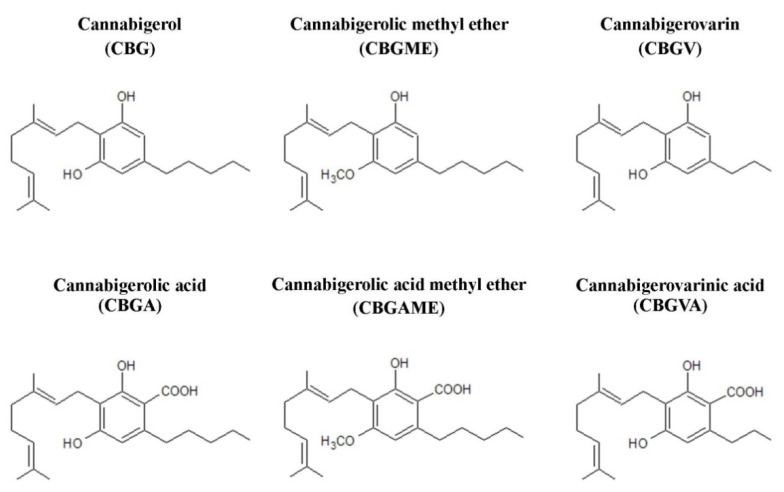
Chemical structures of main compounds from the group of cannabigerol-type phytocannabinoids.

Taking into account their widespread presence in hemp and the significant structural similarity of many phytocannabinoids to cannabigerol (CBG), a separate class of cannabigerol-type compounds has been created (Figure 2). In addition to CBG itself, this group includes cannabigerolic acid (CBGA), CBG, and CBGA methyl ethers, as well as cannabigerovarin (CBGV) and its acid derivative, cannabigerovarinic acid (CBGVA). CBGV is an analogue of CBG containing a three-carbon side chain—in comparison, the side chain of a cannabigerol molecule contains five carbon atoms [16].

The structure of CBG consists of 21 carbon atoms (22 carbon atoms in the case of the acidic form) [25], with the total chemical formula of C_21_H_32_O_2_ and a molar mass of 316.48 g/mol. According to the IUPAC nomenclature, the systematic name of cannabigerol is 2-[(2E)-3,7-dimethylocta-2,6-dienyl]-5-pentylbenzene-1,3-diol. The compound was first isolated in 1964 from a hexane extract of hashish. Its structure and stereochemistry were subsequently confirmed through chemical synthesis [26,27].

The melting point of cannabigerol and its thermal degradation were determined using differential scanning colorimetry and thermogravimetric analysis. It was noted that CBG melts at 52 °C (in comparison, the melting point of cannabidiol is 68 °C) [28]. It was also observed that thermal degradation of cannabigerol occurs at approximately 150 °C (CBD thermally degraded at 250 °C). For both CBG and CBD, these temperatures are close to their boiling points [28]. In contrast, other studies have shown that cannabigerol (like Δ^9^-THC) exhibits greater solubility in supercritical CO_2_ as the temperature increases (at a constant pressure), while cannabidiol shows the opposite trend [29].

## 4. Biosynthesis of Cannabigerol

**Figure 3:**
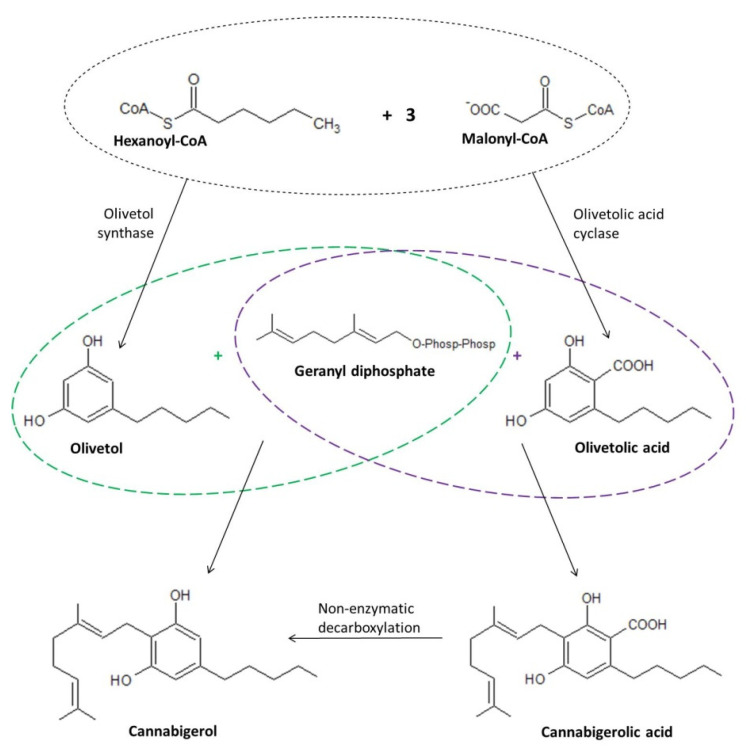
Biosynthesis of CBG including both direct synthesis and the formation of cannabigerol by non-enzymatic decarboxylation of cannabigerolic acid [30,31].

Cannabinoids present in plants are formed by biosynthesis from precursors in the form of the respective fatty acids or geranyl diphosphate (GPP) present within trichomes [13], with two possible mechanisms suggested as those leading to the formation of CBG (Figure 3). In the first mechanism, biosynthesis of cannabigerol occurs by direct synthesis from GPP and olivetol (1,3-dihydroxy-5-pentylbenzene; OL), similarly to the synthetic production of CBG [30]. However, a more likely mechanism of CBG formation is thought to be the process of non-enzymatic decarboxylation of cannabigerolic acid (CBGA) formed from olivetolic acid (OLA) and geranyl diphosphate [8]. This approach seems more realistic due to the fact that olivetol is not detected in *Cannabis sativa* L. tissues despite the presence of olivetol synthase (OLS), an enzyme essential for its biosynthesis [31]. Another factor supporting the latter described mechanism of biosynthesis of cannabigerol is the fact that decarboxylation of CBGA and other phytocannabinoids synthesized in the acid form occurs by non-enzymatic means, including those occurring as a result of long-term storage, or under the influence of irradiation (from either sunlight and UV light) or increased temperature. At the same time, the lack of involvement of enzymes necessary for such a conversion explains the mode of action of orally ingested *Cannabis sativa* L. in the form of heat-treated products (e.g., during the smoking of hemp cigarettes) [30].

**Figure 4:**
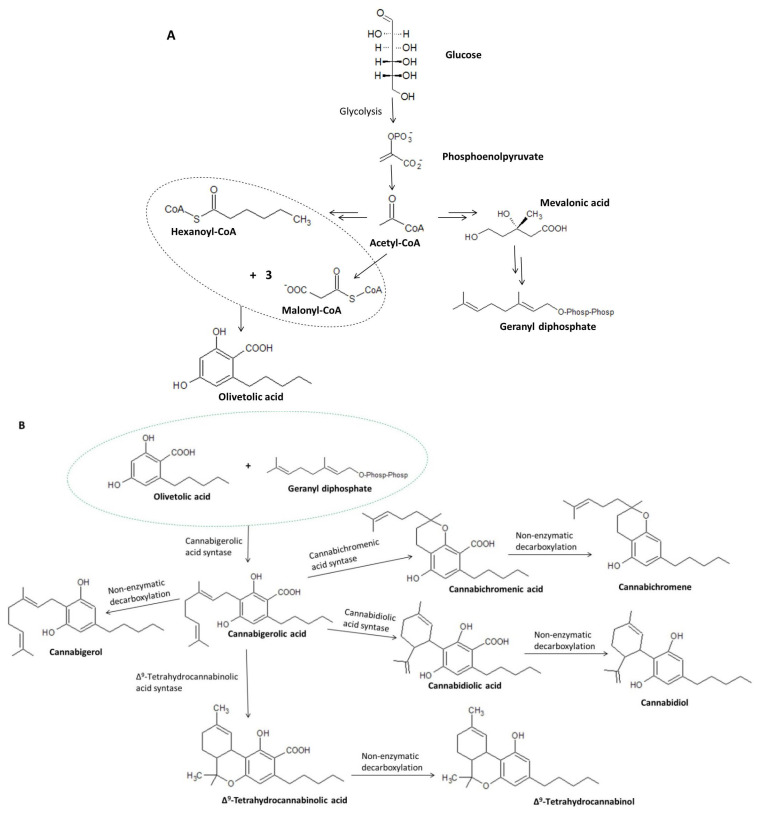
Biosynthesis of olivetolic acid and geranyl diphosphate (**A**) and their further transformations leading to the formation of cannabigerolic acid, including the conversion of CBGA to cannabigerol, cannabidiolic acid, cannabichromenic acid, and Δ^9^-tetrahydrocannabinolic acid (**B**) [8,30,31].

Taking into account the details concerning cannabigerol biosynthesis, it was found that glucose produced through photosynthesis is converted to phosphoenolpyruvate, which—under the influence of pyruvate kinase and pyruvate dehydrogenase—is converted to acetyl-coenzyme A (acetyl-CoA) (Figure 4A). Acetyl-CoA is a key chemical that acts as a substrate in the formation of both geranyl diphosphate and malonyl-CoA, which are necessary for the formation of olivetolic acid and olivetol, i.e., the precursors of cannabigerolic acid [32] and cannabigerol, respectively [31]. Hexanoyl-CoA, on the other hand, is formed during the action of hexanoyl-CoA synthase on hexanoic acid formed in the process of biosynthesis of fatty acid [33]. The aldol condensation of hexanoyl-CoA and malonyl-CoA (ratio 1:3) in the presence of olivetolic acid cyclase (OLAC) results in the formation of olivetolic acid [32], whereas in the presence of olivetol synthase (OLS), decarboxylation with simultaneous cyclization results in the formation of olivetol [31]. As a result of numerous transformations, Acetyl-CoA is initially converted to mevalonic acid (MVA), which is then converted to geranyl diphosphate under the influence of kinases, decarboxylases, and isomerases [34,35].

Under the action of the respective enzymes (THCAS tetrahydrocannabinolic acid synthase and CBDAS cannabidiolic acid synthase), the resulting cannabigerolic acid is converted to Δ^9^-tetrahydrocannabinolic acid and cannabidiolic acid (Figure 4B), which are then decarboxylated to form Δ^9^-THC and CBD, respectively [36,37]. Due to its easy conversion to CBD, cannabigerol is treated as a structural analogue of CBD with an open cyclohexylene ring [38].

The content of cannabigerol in *Cannabis sativa* L. is very low (up to approx. 10% of the cannabinoid fraction) [39], which stems from the fact that some of the cannabigerolic acid present in the plant is converted to acidic forms of other phytocannabinoids from the aforementioned group [12]. Studies of the properties of cannabigerol extracted from *Cannabis sativa* L. are only possible owing to the prior modification of the cannabis genotype aimed at reducing the activity of CBGA-converting synthases to CBDA, Δ^9^-THCA, and CBCA, increasing the CBG content up to 90% of the cannabinoid fraction [39].

## 5. Biological Activity of Cannabigerol

Although cannabigerol is not one of the psychoactive compounds, it exhibits a number of therapeutic properties, including antibacterial, antifungal, and anti-inflammatory effects; it also prevents cell proliferation [40,41]. Similar to cannabinoids, it is believed that cannabigerol’s complex biological effects are the result of modifications of dependent redox and inflammatory processes, which in turn modulate cellular metabolism.

Previously, cannabigerol has been shown to regulate redox balance by reducing the activity of one of the main pro-oxidant factors, i.e., iNOS—activating the membrane receptor PARP-γ—and by modulating the expression of the superoxide dismutase SOD-1, whose activity is increased by pro-inflammatory factors (e.g., lipopolysaccharide, LPS). Consequently, CBG contributes to the inhibition of cell death by shifting the redox balance in the direction of the antioxidant [42]. The phytocannabinoid can also modify inflammatory processes by significantly reducing Iκβ-α phosphorylation, thus reducing the transcriptional activity of the nuclear factor NFκB, responsible for the transcription of pro-inflammatory cytokines [43], which results in reduced levels of cytokines, including TNFα and IL-1β (Table 1) [42].

Based on previous analyses of biological activities of phytocannabinoids, some overlap is believed to exist with the activities of endocannabinoids, especially concerning their actions on G-protein-coupled membrane receptors and lipid mediators as well as phospholipid-metabolizing enzymes [44,45]. Similar to endocannabinoids, phytocannabinoids belonging to the cannabigerol group also modify the activation of CB1 and CB2 cannabinoid receptors as agonists, with CBG also interacting with receptors such as TRPV1 and PPAR [36,46,47]. In addition, cannabigerol decreases the activity of FAAH, an enzyme that metabolizes anandamide, thus affecting its levels and biological effects. However, it should be noted that compared to CBD, CBG is less effective as an FAAH activity inhibitor [48,49]. In contrast, phytocannabinoids from the cannabigerol group, e.g., CBG and CBGA, reduce the activity of DAGL, the enzyme responsible for the biosynthesis of 2-AG and the activities of COX-1 and COX-2, which metabolize PUFAs, mainly arachidonic acid, to lipid mediators [44,50,51]. This causes altered levels of both endocannabinoids and other lipid mediators, which by acting on receptors both directly and indirectly modify both redox balance and inflammation [44]. Consequently, chronic exposure to the bioactive constituents of cannabis leads to decreased CB1 receptor activation, resulting in decreased generation of ROS and TNFα [45]. CBG has a low affinity for CB1 and CB2 receptors (~5-fold and 27-fold lower than Δ^9^-THC), with CBG showing a higher affinity for CB1 [36,52]. The lower affinity of cannabigerol for CB1 compared to other phytocannabinoids explains the lack of psychotropic effect in the case of this particular phytocannabinoid [53]. Cannabigerol intermediates also show the ability to bind to cannabinoid receptors. It is known that CBGA, like CBG, is an agonist of CB1 and CB2 receptors [21], unlike olivetol (OL) which acts as an antagonist of CB1 and CB2 receptors [54]. Cannabigerol exhibits a significant activity against several receptors from the TRP superfamily, including acting as a strong TRPA1 agonist (TRP ankyrin type 1) and a weak TRPV1 agonist (TRP vanilloid type 1), and an even weaker agonist of TRPV2 and TRPV4. It is also a potent inhibitor of TRPM8 (TRP melastatin type 8) [36,49,55]. In addition, CBG has been shown to act as a potent agonist of α2 adrenergic receptor and to moderately block 5-HT1A receptors [52,56], which may explain its biological activity considering its slight affinity for cannabinoid receptors [36].

By influencing the functioning of the endocannabinoid system, cannabigerol modulates many processes within the body, including immune responses, cancer formation, cardiovascular diseases, and pain perception [41]. By inhibiting anandamide uptake, CBG increases anandamide levels and its action in cells [49]. The neuromodulatory effect observed in terms of modifications in the activity of components of the endocannabinoid system is considered particularly important [7]. The same study reported the ability of CBG to reduce anandamide metabolism. In addition, the ability of CBG to inhibit the proliferation of cancer cells (in breast, prostate, colorectal cancers, and gastric adenocarcinoma) through the activation of TRPV1 receptor has been reported [71]. On the other hand, cannabigerol significantly reduces the apoptosis of transformed tumor cells by modulating the levels of Bax and Bcl2 proteins [42], and may thus enhance the development of tumor processes.

Moreover, CBG and its derivatives (both natural and synthetic) have been tested in recent years in terms of their potential use in alleviating the negative effects of chemo-therapy, the treatment of mood disorders (including depression), neurodegenerative diseases, and diseases of the nervous system, and for their anesthetic effects [56,57,70].

## 6. Pharmacokinetics of Cannabigerol

**Figure 5:**
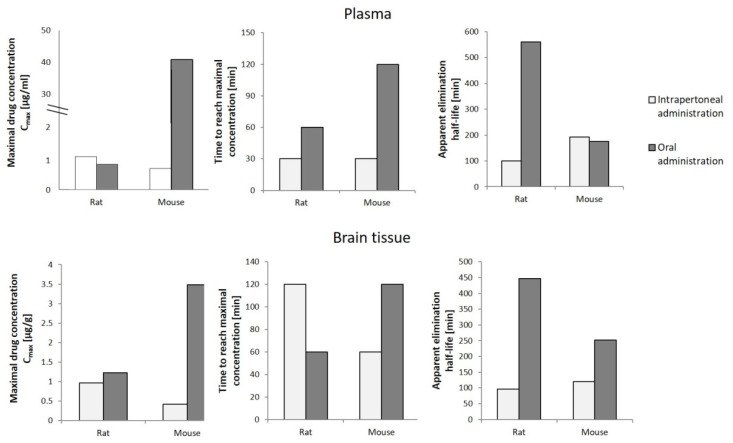
Pharmacokinetics of cannabigerol (120 mg/kg) in rats and mice depending on the mode of administration [72].

To determine the potential use of cannabigerol in pharmacotherapy, in addition to the knowledge about its effect on the metabolic processes occurring in the human body under physiological and pathophysiological conditions, it is necessary to analyze the pharmacokinetics of the compound in question after its introduction into the body. In vivo studies have shown that cannabigerol suspended in mixtures of glycerol and ethylene oxide (cremophor EL) and ethanol and saline in a ratio of 1:1:18 administered orally or intraperitoneally to rats and mice in varying amounts concentrates in the blood plasma [72]. It has been found that the plasma concentration of CBG in rats was slightly higher after intraperitoneal administration compared to the concentration reached after oral administration (Figure 5). In contrast, the maximum plasma concentration of CBG in mice was over 60 times higher after oral administration compared to intraperitoneal administration, yet this level was reached as late as after 2 h (in the case of intraperitoneal administration, the maximum concentration was reached after 30 min). In addition, brain levels of CBG in both mice and rats were shown to be higher in the case of intraperitoneal administration, which favors very significant increases in CBG levels. The studies also showed that intraperitoneal administration was accompanied by a longer elimination half-life of cannabigerol, with no common relationship between the mode of administration of CBG and the time required to reach its maximum concentration in both animal models tested [72].

Determination of CBG concentrations in the human body, mainly in the blood, is also used for diagnostic purposes, i.e., as a biomarker of recent use of cannabis containing the narcotic Δ^9^-THC. This is all the more important due to the fact that, according to data published in the 2021 World Drug Report, hemp (including *Cannabis sativa* L.) is the most widely used drug worldwide. According to the report, it is estimated that almost 4% of the global human population has used cannabis at least once in 2019 alone, which equates to around 200 million people [73]. Given past trends, it can be assumed that this number will continue to rise in the coming years. Thus, knowledge of the pharmacokinetics of the phytocannabinoids present in *Cannabis sativa* L., obtained using different types of biological material, may be necessary to assess the intake of *Cannabis sativa* L.-based preparations, as well as the time of their use.

**Figure 6:**
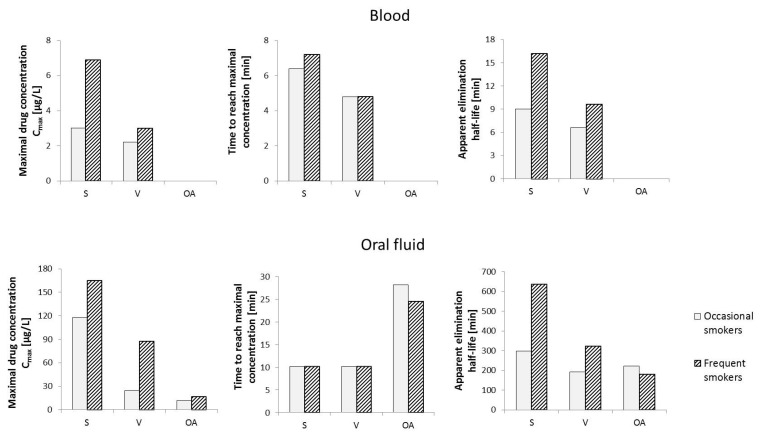
Changes in cannabigerol levels in human blood and oral fluid over time depending on the route of administration of *Cannabis sativa* L. Abbreviation: S—smoking, V—vaporization, OA—oral administration [74,75].

Considering that in the case of frequent cannabis smokers, Δ^9^-THC can be detected in the blood as late as 30 days after the last use, the phytocannabinoid cannot be used to determine the exact time when *Cannabis sativa* L. was smoked. Due to the changes in CBG levels in the living body, including in the blood, over a relatively short period of time after exposure to the compound, the variation in cannabigerol concentrations after vaporization or smoking *Cannabis sativa* L. was studied (Figure 6). In smokers of cannabis in the form of “active” cigarettes, maximum CBG concentrations were found to reach 6.9 μg/L after about 7 min and 3.0 μg/L after about 6 min in frequent and occasional smokers, respectively. It was found that detection of cannabigerol in the blood of frequent smokers is possible up to 16 min after smoking an active cigarette, while in occasional smokers this time is reduced to 9 min. In the case of vaporization, lower levels of mean maximum CBG concentrations in blood were observed in both study groups, which can be explained by the lower efficiency of release of cannabigerol from *Cannabis sativa* L. under conditions of vaporization, compared to smoking. In contrast, no cannabigerol was detected in the blood of subjects who were administered *Cannabis sativa* L. orally [74].

In contrast, other studies have reported that cannabigerol was only sporadically present (in approx. 2% of the samples tested) in plasma obtained from individuals participating in several clinical trials focused on the effects of medical marijuana on various pathological conditions (e.g., Parkinson’s disease, brain tumors in children, childhood epilepsy). It was observed that the samples in which CBG was detected showed a significant Δ^9^-THC content, indicating that medical marijuana had been consumed shortly before the study took place [76]. The above data, therefore, suggests that monitoring cannabigerol levels in the blood may constitute an effective marker to confirm the fact of recent cannabis smoking.

A similar evaluation of the effect of the mode of administration of *Cannabis sativa* L. on oral fluid levels of cannabigerol showed that when *Cannabis sativa* L. is taken orally, the maximum mean CBG content is the lowest in both occasional users and frequent smokers (11.9 μg/L and 17.0 μg/L, respectively). Interestingly, in terms of the mode of administration, of the three analyzed routes, the time required to reach the maximum CBG concentration in oral fluid is the longest in the case of smoking (approximately 28 min and 25 min for occasional and frequent smokers, respectively). The highest concentration values (C_max_) in individuals in the study groups were again observed when “active” cigarettes were smoked (165 μg/L and 118 μg/L, for occasional and frequent smokers, respectively). In contrast, the time required to reach maximum CBG concentrations in the analyzed oral fluid samples was about 10 min for both occasional and frequent smokers. Hence, cannabigerol introduced into the body by smoking *Cannabis sativa* L. persists in the body for much longer than in the case of other forms of cannabis use (over 10 h on average for frequent smokers and nearly 5 h for occasional smokers) and reaches significantly higher levels, especially in oral fluid, compared to blood [75].

Considering the pathway of CBG biosynthesis in a living organism, the presence of both CBG and its precursor, i.e., cannabigerolic acid (CBGA), was tested in the blood plasma of patients in addiction treatment units by analyzing the presence of phytocannabinoids at different times after *Cannabis sativa* L. was ingested [77]. CBG was found in 22 out of 56 subjects at 24 h and in 4 out of 17 subjects at 26.4–73 h after ingestion. In contrast, CBGA was only present in 12 out of 56 subjects (after 24 h) and in 1 in 17 subjects (after 26.4–73 h). The mode of ingestion of *Cannabis sativa* L. (smoking or oral ingestion) was not taken into account in the study, nor was it possible to check whether the ingested cannabis had undergone heat treatment [77].

Considering these observations, it can be concluded that the determination of cannabigerol levels in oral fluid makes it possible to estimate the time of ingestion of orally administered cannabis-based preparations. A simultaneous analysis of both blood and oral fluid suggests that it is possible to obtain information not only about the time elapsed since the ingestion but also on how *Cannabis sativa* L. was introduced into the body.

In addition, it has been shown that it is possible to determine urinary cannabigerol levels in *Cannabis sativa* L. smokers. The compound is absorbed into the body during smoking, where it is metabolized and then excreted in the urine [78]. It was observed that cannabigerol was removed from the bodies of *Cannabis sativa* L. smokers in urine in the form of a conjugate with glucuronic acid, which was not found in either the control or the CBG-enriched urine samples. The absence of this compound in these samples indicates that it is not produced at the stage of preparation of samples for analysis, and its presence in the analyzed samples can only result from CBG metabolism [79].

## 7. Summary

At the moment, cannabigerol is one of the least-known phytocannabinoids found in *Cannabis sativa* L., which, however, shows promising potential in therapeutic actions. Considering that both CBG and its precursors and metabolites are lipophilic, it favors the penetration the penetration through biological membranes and indicates the possibility of biological activity in the lipid sphere mainly through interactions with the endocannabinoid system, including G-protein-coupled receptors. As a result of these interactions as well as direct actions, CBD exhibits antioxidant and anti-inflammatory properties, while both CBG and CBGA as well as its synthetic derivatives exhibit neuromodulatory effects. Moreover, CBG has been shown to reduce the survival of glioblastoma cells, similar to temozolomide used both in monotherapy and with CBG [80]. So far, however, the data in this regard are inconclusive and, moreover, come from in vitro and animal studies that require validation on human tissues and cells used ex vivo, prior to possible clinical trials. No harmful effect of CBG on the human body has been found so far, and the proven biological activity indicates CBG and its derivatives as very promising natural compounds that should be thoroughly tested both in vitro and in vivo in order to unequivocally determine the therapeutic usefulness, especially with regard to inflammatory diseases. Therefore, it seems obvious that new therapeutic approaches using the non-psychoactive ingredients of *Cannabis sativa* L, including CBG, can be expected in the nearest future.

## Figures and Tables

**Table 1:** Biological activity of CBG and its derivatives.

	CBG	CBGA	VCE-003	VCE-003.2
Activity Area				
**Receptors**	Agonist: CB1, CB2, PPARγ, TRPA1, TRPV1, TRPV2, TRPV3, TRPV4, α2-adenoceptors	Agonist: CB1, CB2, PPARγ, TRPA1, TRPV1, TRPV2, TRPV3, TRPV4	Agonist: CB1, CB2, PPARγ	Agonist: CB1, CB2, PPARγ
	Antagonist: TRPM8, 5-HT_1A_	Antagonist: TRPM8		
**Endocannabinoid system enzymes**	Inhibition: FAAH, DAGLα, MAGL	Inhibition: FAAH, MAGL, DAGLα	Inhibition: MAGL	Inhibition: FAH, MAGL
**Redox status and inflammation**	Downregulation: TNFα, NFκB, IL-1ß, IL-6, INF-γ, PGE_2_	Downregulation: TNFα, NFκB, IL-1ß, IL-6, INF-γ, PGE_2_	Downregulation: TNFα, IL-1ß, IL-2, IL-6, IL-17, INF-γ, PGE_2_	Downregulation: TNFα, NFκB, IL-1ß, IL-6, PGE_2_, Caspase 3
	Inhibition: iNOS, COX-1, COX-2, PLA2	Inhibition: iNOS, COX-1, COX-2, PLA2	Inhibition: iNOS,	Inhibition: iNOS, COX-2
	Upregulation: Catalase, SOD-1		Upregulation: Nrf2	
**Biological activity**				
	antioxidant anti-inflammatory antibacterial neuromodulatory neuroprotective	antioxidant antibacterial neuromodulatory neuroprotective	anti-inflammatory neuromodulatory neuroprotective	anti-inflammatory neuromodulatory neuroprotective
**References**				
	[21,57,58,59,60,61,62,63,64]	[21,49,58,59,62,65]	[57,64,66,67,68,69,70]	[57,63,64,66,68]

Abbreviation: CBG—cannabigerol; CBGA—cannabigerolic acid; VCE-003—cannabigerol quinone; VCE-003.2—second-generation cannabigerol quinone derivative.

## Data Availability

Not applicable.
